# LIFE LESSONS: THE EXPERIENCES OF OLDER ADULTS LIVING IN PUERTO RICO DURING COVID-19

**DOI:** 10.1093/geroni/igac059.623

**Published:** 2022-12-20

**Authors:** Humberto Fabelo, Matthew Morgan, Julia Vazquez, Todd Becker

**Affiliations:** Virginia Commonwealth University, Richmond, Virginia, United States; Virginia Commonwealth University, Richmond, Virginia, United States; University of Maryland, Baltimore, Baltimore, Maryland, United States; University of Maryland, Baltimore, Baltimore, Maryland, United States

## Abstract

What life lessons emerge from a group of older Puerto Ricans concerning their experiences with COVID-19. We will review study participants’ reports of how they spent their time during the pandemic, what they found most difficult to cope with, and life lessons they learned as a result. We will use Erickson’s life course theory of psychosocial development to frame their responses to these questions, including the crises (and strengths) of generativity versus stagnation (care) and ego integrity versus despair (wisdom). We will report evidence of care and wisdom as core ego strengths resulting from their lived experience. We will also review how isolation, sense of community, and key mental and physical health factors seemed to influence how their responses suggest coping strengths. We will discuss our findings in the context of social and cultural norms of current cohorts of older Puerto Ricans, highlighting the salience of community and family relations. (150 words)

